# Genome-Wide Association Studies of Somatic Cell Count in the Assaf Breed

**DOI:** 10.3390/ani11061531

**Published:** 2021-05-24

**Authors:** Yasemin Öner, Malena Serrano, Pilar Sarto, Laura Pilar Iguácel, María Piquer-Sabanza, Olaia Estrada, Teresa Juan, Jorge Hugo Calvo

**Affiliations:** 1 Department of Animal Science, University of Uludag, Bursa 16059, Turkey; onery@uludag.edu.tr; 2 Departamento de Mejora Genética Animal, INIA, 28040 Madrid, Spain; malena@inia.es; 3 Unidad de Producción y Sanidad Animal. Centro de Investigación y Tecnología Agroalimentaria de Aragón —Instituto Agroalimentario de Aragón (IA2) (CITA—Zaragoza University), 50059 Zaragoza, Spain; mpsarto@aragon.es (P.S.); lpiguacel@cita-aragon.es (L.P.I.); mariamooneyes@gmail.com (M.P.-S.); oestradak@gmail.com (O.E.); tjuan@cita-aragon.es (T.J.); 4 ARAID, 50018 Zaragoza, Spain

**Keywords:** dairy, sheep, GWAS, mastitis, somatic cell count

## Abstract

**Simple Summary:**

Mastitis causes economic loss due to discarded milk and reduced milk production and quality, increased medical care costs and somatic cell count (SCC) penalties. The use of genetic markers associated with the variability of this trait through marker-assisted selection (MAS) could help traditional methods. Our objectives were to identify new single nucleotide polymorphisms (SNPs) and genes associated with mastitis resistance in Assaf sheep by using the Illumina Ovine Infinium® HD SNP BeadChip (680K). Firstly, corrected phenotype estimates for somatic cell score (SCS) were calculated using 6173 records from 1894 multiparous Assaf ewes, and were used to select 192 extreme animals (low SCS group: n = 96; and high SCS group: n = 96) for the genome-wide association study (GWAS). Four SNPs (rs419096188, rs415580501, rs410336647, and rs424642424), three of them totally linked, were found to be significant at the chromosome level (FDR 10%) in two different regions of OAR19 close to genes related to the immune system response. Validation studies of two SNPs (rs419096188 and rs424642424) by Kompetitive Allele-Specific PCR (KASP) genotyping in the total population (n = 1894) confirmed previous GWAS association results for the SCS trait. Finally, the SNP rs419096188 was also associated with lactose content trait.

**Abstract:**

A genome-wide association study (GWAS) was performed to identify new single nucleotide polymorphisms (SNPs) and genes associated with mastitis resistance in Assaf sheep by using the Illumina Ovine Infinium^®^ HD SNP BeadChip (680K). In total, 6173 records from 1894 multiparous Assaf ewes with at least three test day records and aged between 2 and 7 years old were used to estimate a corrected phenotype for somatic cell score (SCS). Then, 192 ewes were selected from the top (*n* = 96) and bottom (*n* = 96) tails of the corrected SCS phenotype distribution to be used in a GWAS. Although no significant SNPs were found at the genome level, four SNPs (rs419096188, rs415580501, rs410336647, and rs424642424) were significant at the chromosome level (FDR 10%) in two different regions of OAR19. The SNP rs419096188 was located in intron 1 of the *NUP210* and close to the *HDAC11* genes (61 kb apart), while the other three SNPs were totally linked and located 171 kb apart from the *ARPP21* gene. These three genes were related to the immune system response. These results were validated in two SNPs (rs419096188 and rs424642424) in the total population (*n* = 1894) by Kompetitive Allele-Specific PCR (KASP) genotyping. Furthermore, rs419096188 was also associated with lactose content.

## 1. Introduction

Mastitis is the most frequent inflammation-driven disease that occurs in response to infection with pathogenic microorganisms such as *Escherichia coli, Streptococcus uberis,* and *Staphylococcus aureus* [1] or physical damage. This disease is associated with substantial economic losses for the sheep dairy sector due to the cost of discarded milk, reduced milk production and quality, and increased medical care costs. Furthermore, the disease may also lead to the spread of zoonotic diseases and the progression of resistance to antibiotics.

In Spain, dairy sheep are used mainly for cheese making and often have a regional or local identity regarding origin and quality [2], which plays an important economic role in less-favoured rural regions. Spanish Assaf, currently the most important dairy sheep breed in Spain, is a synthetic crossbreed (Awassi × east Friesian) originating in Israel; there are currently 145,000 Assaf ewes in Spain. The main objective of the genetic selection program of the Assaf sheep breed is to increase milk yield and quality (protein and fat content). However, traits related to milk health based on the somatic cell count (SCC) as an indicator trait for mastitis resistance and udder morphology have also been included since 2017.

Studies of genetic parameter estimation have revealed that in addition to environmental conditions and pathogens, the severity of mastitis depends on the host response [3]. The response of animals to infection is heritable [4], although heritability estimates of the trait are moderate. In the Spanish Assaf sheep breed and other breeds, the heritability estimated for SCS was around 0.16 [3,5]. In this sense, the use of genetic markers associated with the variability of this trait through marker-assisted selection (MAS) could help traditional methods [6,7] to increase the genetic response in the framework of the breeding program to improve mastitis resistance. Furthermore, the use of molecular marker information is known to increase the accuracy of estimated breeding values (EBVs) and reduces the rate of pedigree-estimated inbreeding [8,9]. Although mastitis is one of the most frequent infectious diseases in dairy husbandry and has been intensively studied in cattle [10,11,12], studies in dairy sheep are scarce. Since 2007, when the sheep genome was first sequenced, the development of low- and high-density ovine single nucleotide polymorphisms (SNP) chips [13] has allowed genome-wide association studies to reveal the genetic background of several diseases, including mastitis susceptibility in sheep [14,15,16,17]. These studies have highlighted several genome regions and some candidate genes, some of which are immune- and body growth-related, distributed on different chromosomes [16,17,18,19]. Despite this challenge in sheep, genome-wide association studies for mastitis have remained limited [3].

For the reasons stated above, an improved breeding scheme with genetic markers can allow one to cope with the disease. Therefore, the main objective of this study was to identify new SNPs and genes associated with mastitis resistance in Assaf sheep by using the Illumina Ovine Infinium® HD SNP BeadChip (680K) in extreme phenotype animals for the somatic cell score (SCS) trait (*n* = 192) and to validate some of the significant SNPs in the total population used to characterize the SCS trait (*n* = 1894). 

## 2. Materials and Methods

### 2.1. Animals

Three flocks of the Spanish Assaf breed belonging to the Teruel Association of Dairy and Cheese Producers, which was established with the aim of promoting artisan cheese production, were selected for this study. In total, we used 1894 multiparous ewes between 2 and 7 years old with two or more lactations and with at least 3 test day records during one lactation: flocks A (*n* = 574), B (*n* = 893), and C (*n* = 427). A total of 6173 records for the SCC, fat (FC), protein (PC), lactose (LC), and total solid content (TSC) from 1894 ewes were considered. For milk yield (MY), 2697 records were obtained from 1001 ewes because no MY data were obtained from flock B. The SCC data were logarithmically transformed into somatic cell scores (SCS = (log2 (SCC/100,000) + 3)) [20].

Corrected phenotypes for the somatic cell score (SCS) trait were calculated (Section 2.3.1) for the 1894 ewes, and 192 ewes were selected from the high (*n* = 96) and low (*n* = 96) tails of the corrected SCS phenotype distribution for the genome-wide association study (GWAS). The ewes selected for these groups were in the highest (H) 10% and lowest (L) 14% tails of the distribution of corrected phenotypes obtained for all ewes. Sixty-four ewes from each flock (*n* = 32 for low SCS; and *n* = 32 for high SCS) were selected as unrelated as possible based on pedigree records existing within herds. The GWAS results were validated by genotyping two of the significant SNPs in the GWAS in 1824 ewes belonging to the three flocks (A: *n* = 510; B: *n* = 891; C: *n* = 423) from the total population (*n* = 1894). Seventy ewes were not taken into account because it was not possible to obtain a blood sample.

### 2.2. Genotyping

#### 2.2.1. GWAS

Genomic DNA was extracted from blood samples using the FlavorPrep Genomic DNA mini kit (Flavorgen, Ibian, Zaragoza, Spain). Genotyping of 192 ewes was performed with the Ovine HD SNP BeadChip (Illumina, Inc., San Diego, CA, USA) designed by the International Sheep Genome Consortium [21]. SNP genotyping services were provided by the Spanish **“**Xenetica Fontao” company (https://www.xeneticafontao.com; accessed on 19 April 2021).

#### 2.2.2. SNP Genotyping in GWAS Validation

Genomic DNA was extracted as described above. Based on the results of the GWAS, two SNPs located in OAR19 were selected for validation studies (rs419096188 and rs424642424). These SNPs were genotyped using fluorescent Kompetitive Allele-Specific PCR (KASP). The sequence surrounding the target polymorphism was used to design primers for genotyping by the platform provider (LGC Genomics, Teddington, UK). KASP reactions were carried out following the manufacturer’s protocol. For all samples, KASP assays were performed in a 10 μL volume containing 1 µL of DNA (20 ng), 5 μL of KASP V4.0 2x Master mix standard ROX (LGC Genomics, UK), and 0.14 μL of KASP-by-Design assay mix (LGC Genomics, UK). Reactions were carried out in a CFX96 BioRad thermocycler (BioRad, Madrid, Spain) using the following conditions for both pairs of primers: 94 °C for 15 min followed by 9 touchdown cycles of 94 °C for 20 s and 57 °C for 60 s (de-creasing −0.6 °C per cycle) followed by 25 additional cycles of 20 s at 94 °C and 60 s at 55 °C. Following PCR, fluorescence was detected using a single quantification cycle for 1 s after cooling at 30°C for 2 min.

### 2.3. Statistical Analysis

#### 2.3.1. Corrected Phenotype Values for the Somatic Cell Score (SCS) Data

To obtain a corrected phenotype for SCS to be used in the GWAS analyses, a linear mixed model using the high-performance mixed procedure (HPMIXED) in SAS (Version 9.3; SAS Inst. Inc., Cary, NC, USA) was performed. The model included the herd-test day (HTD), lactation number (LN), and the number of lambs born (Nlb) as fixed effects; the days in milk (Dim) and the age (A) as covariates; and the permanent effect of the animal (P) to which repeated records belong and the residual (e) as random effects. Homogeneous variances were considered for the permanent effect [P ~ N (0, σ2)] and the residual [e ~N (0, σ2)]. Corrected phenotypes estimates adjusted by the factors included in the model were used to select 192 animals from the upper and bottom tails of the corrected phenotypes distribution of the whole population (*n* = 1894) to be used in the GWAS.

#### 2.3.2. Quality Control (QC)

We applied the QC criteria to the raw genotypes as follows: (i) individuals with low call rates (<0.97) were excluded from additional analysis; and (ii) SNPs were also excluded if they had a low call rate (<0.97), a minor allele frequency (MAF) < 0.01, or significant deviations from Hardy-Weinberg equilibrium (HWE) (*p*-value < 0.00001). QC was performed using PLINK 1.9 [22].

#### 2.3.3. Stratification Analysis

Pair-wise linkage disequilibrium (LD) measured by the r^2^ threshold for the population was calculated for each chromosome using all SNPs that passed QC by PLINK 1.9 [22]. All autosomal SNPs were pruned using the “indep-pairwise” option, with a window size of 50 kb, a step of 10 SNPs, and an r^2^ threshold of 0.2, resulting in 82,876 independent SNP markers. Clustering and multidimensional scaling (MDS) analyses were performed on genome-wide identity-by-state (IBS) pair-wise distances to check outliers and population stratification and the appropriate choice of high- and low-SCS animals within the population. Plotting the component C1 values against component C2 allows us to identify clustering of samples using standard classical (metric) multidimensional scaling.

#### 2.3.4. Genome-Wide Association Analyses (GWAS)

The GWAS for estimated SCS data was performed using the mixed linear model-based association analysis (MLMA) of the genome-wide complex trait (GCTA) analysis software [23] for the whole genome and included a genetic relationship matrix to control for the random effects of genetic similarity, excluding the chromosome on which the candidate SNP is located (leaving-one-chromosome-out LOCO), by applying the following model:

yj = u + SNPi + gj+ ej

where yj is the corrected phenotype for SCS for the genotyped animal j, u is the overall mean, SNP is the effect of the i SNP (assumed as a covariate coded as 0, 1 or 2, respectively, to genotypes aa, Aa and AA), gj is the random additive genetic effect, and ej is the residual error. The significance of association will be assessed using a false discovery rate (FDR) multitest correction threshold. Chromosome-wide significance association was assessed using an FDR = 0.1 multitest correction threshold. The threshold value of 10% was selected because we decided to be more conservative. Visualization of the association data in Manhattan plots and quantile-quantile plots was performed using SNPEVG software [24]. The number of false positives was controlled by calculating the genomic inflation factors as the observed median χ2 divided by the expected median χ2. The positional candidate genes were identified in the 250 kb region on both sides of the significant SNPs according to the sheep genome assembly (Oar_v3.1) and based on Ensembl release 81.

#### 2.3.5. Gene Association Analysis

The Hardy–Weinberg equilibrium exact test values, observed and expected heterozygosities, and minor allele frequency (MAF) for each SNP were calculated using PLINK 1.9 software [22]. The relationships between the SNPs rs419096188 and rs424642424 and the SCS were estimated by fitting a linear mixed model using the high-performance mixed procedure (HPMIXED) of SAS (Version 9.3; SAS Inst. Inc., Cary, NC, USA). The model included the herd-test day (HTD), the number of lambs born (Nlb), lactation number (LN), and the genotype (G) as fixed effects; the days in milk (Dim) and the age (A) as covariates; and the permanent effect of the animal (P) from which records were collected and the residual (e) as random effects. The equation of the model was as follows:

y(SCS) = Nlb + HTD + G + LN + b (Dim) + b (A) + P + e

To test differences between genotypes, the least squares means (LSMs) for each pair-wise comparison were estimated.

In addition, the relationships between the SNPs rs419096188 and rs424642424 and MY, PC, FC, LC, and SC were also estimated as described above.

## 3. Results

### 3.1. Phenotype and SNP Data of GWAS

Corrected phenotype estimates for the somatic cell score (SCS) were calculated for all ewes considered in this study (*n* = 1894). The corrected phenotype obtained was the permanent effect of each ewe, which is a new phenotype without the known effects. Table 1 shows the individual estimated mean values for the GWAS selected ewes within high and low SCS tails.

The GWAS was performed after the QC of the raw genotypes. Any sheep were removed for a call rate less than 97%. A total of 559,762 SNP markers distributed on the 26 and X ovine chromosomes were included in subsequent analyses.

MDS analysis of 82,876 independent SNPs using the first two MDS components showed that individuals clustered together within each flock. However, some animals from the C flock clustered within the A flock cluster (Figure S1). No phenotype stratification was found when the high and low SCS animals from the three flocks were plotted (Figure S1b).

### 3.2. Genome-Wide Association Analyses (GWAS)

**Figure 1:**
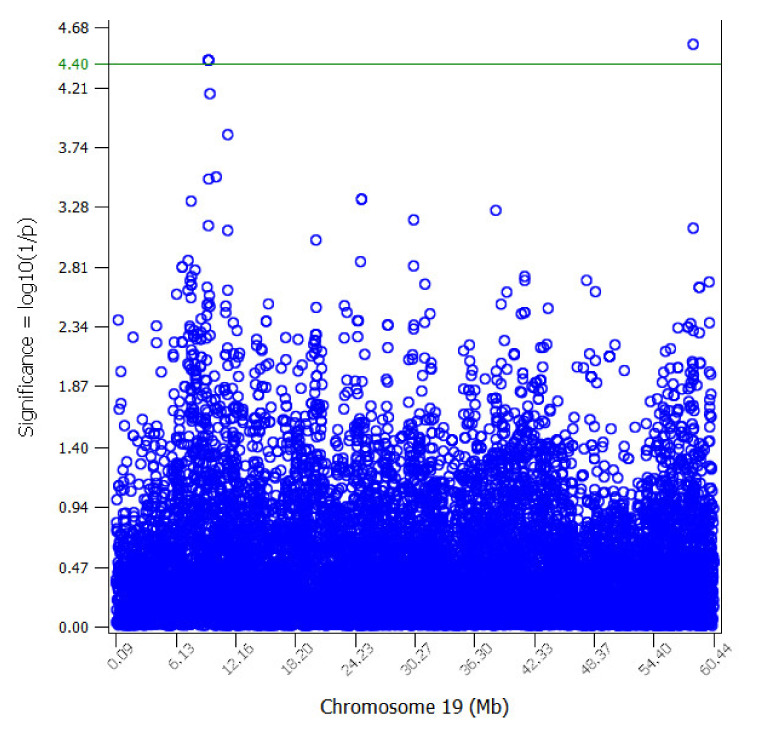
Manhattan plot of chromosome-wide association study of the corrected phenotype of somatic cell score (SCS) trait in Assaf sheep for chromosome Oar19. The chromosome-wide significance level was established at values above log10 (1/*p*) (observed value) >4.40 (FDR *p* < 0.10).

The genomic inflation factor was used to assess bias in the test statistics. The average genomic inflation factors were 1.012 and 1.016 for MLMA and LOCO approaches, respectively, which suggests that any potential bias due to population stratification was addressed. No significant genome-wide results were found. Figure S2 shows a Manhattan plot across the whole genome for the estimated SCS trait, with SNP associations represented as log10 (1/*p*-value) on the y-axis. However, 4 SNPs in OAR19 were significant at the chromosome level (FDR *p* < 0.10) (rs419096188, rs415580501, rs410336647, and rs424642424) (Table 2, Figure 1). As the MLMA and LOCO approaches yielded similar results for all traits analyzed, only the results for the MLMA approach are shown. Notably, SNPs rs415580501, rs410336647, and rs424642424 were completely linked (r^2^ = 1).

Putative causal genes located in the 250 kb region on both sides of the significant SNPs for the SCS trait are also indicated in Table 2. For SNPs rs415580501, rs410336647, and rs424642424, the *ARPP21* gene was annotated in this interval in OAR 3.1. The SNP rs415580501 was located 171 kb apart from the *ARPP21* gene. Furthermore, a miRNA (*miR-128-2*) is located in an intron of this gene. For the SNP rs419096188, 4 coding genes (*FBLN2*, *Ensoarg00000001587*, *HDAC11*, and *NUP210*) and 3 long noncoding RNAs (*Ensoarg00000026664*, *Ensoarg00000026665*, and *Ensoarg00000026666*) were annotated in the 250 kb interval. The SNP rs419096188 was located in the 5’ region or in intron 1 of the *NUP210* gene according to the OAR 3.1 and Oar_Rambouillet_v1.0 (GCF_002742125.1) genome versions, respectively, while the *FBLN2*, *Ensoarg00000001587*, and *HDAC11* genes were located at 164, 153, and 61 kb, respectively, in Oar3.1.

### 3.3. Validation Studies in the ASSAF Total Population

In total, 1824 ewes belonging to the three flocks were genotyped by KASP for both SNPs. Only one genotype failure in one ewe for rs424642424 was found. The SNPs studied were in Hardy-Weinberg equilibrium. The association studies showed that the HTD and Dim effects were significant for the two SNPs for all traits (Table 3). The genotype effect was significant for both SNPs for the SCS trait and for lactose content for rs419096188. For rs419096188, the SCS from animals carrying the AA genotype was 0.21 ± 0.07 and 0.26 ± 0.08 lower than that from ewes with the AG (*p* = 0.011) and GG (*p* = 0.002) genotypes, respectively. In the case of rs424642424, the SCS showed an increase of 0.16 ± 0.05 in AG ewes compared to GG (*p* = 0.002) animals. These results confirm those obtained by the GWAS. Finally, for rs419096188, the LC from animals carrying the AA genotype was 0.046 ± 0.016% higher than that from ewes with the GG genotype (*p* <0.05).

## 4. Discussion

This study identified two different genomic regions in OAR19 associated with the SCS, a trait that is highly correlated with ovine mastitis and that has high negative effects on farm economy, animal welfare, and milk product health for the dairy industry. According to our findings, no significant SNPs at the genome-wide level were found; however, four SNPs were found to be significant at the chromosome-wide level in OAR19. Three of these SNPs were completely linked (rs415580501, rs410336647, and rs424642424) (Table 2) and were located close to the *ARPP21* gene, which includes a miRNA (*mir128-2*) located in one of its introns. Mammals have two genes for *miR-128* that are located in introns of two conserved, orthologous protein-coding host genes, *R3HDM1* and *ARPP21*, that harbor *miR-128-1* and *miR-128-2*, respectively. The SNP rs419096188 is located in the interval where four coding genes (*FBLN2*, *Ensoarg00000001587*, *HDAC11*, and *NUP210*) and three long noncoding RNAs were annotated (Table 2). The SNP rs419096188 was located in the 5’ region or intron 1 of the *NUP210* gene according to the OAR 3.1 (Texel) or Oar_Rambouillet_v1.0 (GCF_002742125.1) sheep genome versions, respectively, while *FBLN2*, *Ensoarg00000001587*, and *HDAC11* genes were located at 164, 153, and 61 kb, respectively, in Oar3.1.

Different results were obtained in previous GWASs for mastitis resistance in sheep. In Sarda [14], Lacaune [16], Chios [18], Churra [17], and Frizarta [25] dairy sheep breeds, GWASs indicated significant regions located on OAR3, OAR4, OAR5, OAR11, OAR12, OAR16, OAR18, OAR19, OAR20, OAR22, and OAR23. Banos et al. [26] suggested for the first time the involvement of chromosome 19 in mastitis resistance. However, these authors found significant SNPs in a different region (approximately 24 Mb on OAR19) from those detected in our study (approximately 9.5 and 58 Mb on OAR19). Some GWASs have suggested that OAR3 is the most significant chromosome for mastitis resistance [16,18], while others have found OAR20 to be the most associated with this trait [14,27]. The most significant genes associated with mastitis resistance are related to the immune response, but others are involved in growth traits. Among the mutations published, a SNP in the *suppressor of cytokine signaling 2* (*SOCS2*) gene has been shown to be particularly important for the trait being related to the inflammatory response and has been detected in Lacaune [16] and Chios [18] sheep breeds.

In our study, three genes (*ARPP21*, *NUP210*, and *HDAC11*) and one miRNA (*miR-128-2*) could be functionally related to the trait studied. Hosting *miR-128-2* is related to breast and prostate cancer in humans [28]. The mir128-2 gene was grouped together with female-specific tymulus genes in sheep [29]. This gene is highly expressed in the mammary gland, and its expression level depends on the lactation state and infection circumstances in livestock [30,31,32,33]. In this sense, Lawless et al. [32] showed an overexpression of *miR-128-2* in cultured bovine mammary epithelial cells after six hours of infection with *S. uberis*. Recently, Ren et al. [34] described that *miR-128* inhibits the expression of inflammatory cytokines to avoid excessive inflammation by regulating the nuclear factor kappa-light-chain-enhancer of activated B cells (NF-κB) signaling pathway. The *ARPP21* gene was thought to be related to the response to stimulus, triggering a cellular response to heat at any temperature higher than the optimal temperature for the organism [28]. Furthermore, the *ARPP21* gene was downregulated after 2 h of infection with *S. Typhi* [35] in a transcriptome study of human intestinal tissue after early infection. Recently, it has also been suggested that *ARPP21* could antagonize miRNA function in general and *miR-128* targeting in particular [36] since the *ARPP21* gene was upregulated in a mouse *miR-128* knockout [37].

The second coding gene was *HDAC11* (histone deacetylase 11), located near rs419096188 and involved in the immune system process [12]. HDAC11 belongs to the histone deacetylase (HDAC) family and the only class IV histone deacetylase, which are essential regulators of gene transcription in all eukaryotic organisms [38,39]. This gene is considered one of the most important factors in epigenetic mechanisms of the immune system and malignant process regulation. Together with other HDACs, HDACs are involved in processes such as cell differentiation, the DNA damage response, and the cell cycle [40]. *HDAC11* also has important roles in initiating adaptive immune responses, such as type I interferon (IFN) signaling and CD4+ T cells [41]. Finally, the SNP rs419096188 is located on the *NUP210* gene, which is involved in the immune response, cholesterol homeostasis, and stress response. The *NUP210* gene belongs to the nucleoporin (NUP) protein family and is involved in nucleocytoplasmic transport through nuclear pore complexes (NPCs). The expression of this gene has been found to be related to tube morphogenesis and mammary gland development in French dairy cattle breeds [42]. Furthermore, and related to its immune function, NPCs have been found to play a key role in the import of transcription factors in T cells in the context of inflammation and immune processes [43]. Borlido et al. [44] showed that *NUP210* deletion revealed a cell intrinsic role in the regulation of CD4+ T cell homeostasis, specifically reducing the number of circulating naïve CD4+ T lymphocytes and establishing tissue-specific NPCs as key modulators of T cell receptor (TCR) signaling in mice.

The two significant genomic regions associated with mastitis resistance were validated by genotyping one SNP of each region in the whole ewe population (*n* = 1824). The validation results confirmed those of the GWAS. Both SNPs were associated with the SCS trait, and rs419096188 was also significant for lactose content (Table 3). Animals carrying the AA genotype had lower and higher SCS values and lactose content, respectively, than animals with the GG genotype. This result is not surprising because a negative correlation has been shown between the SCS and lactose content in other studies. For example, Pazzola et al. [45] found negative phenotypic and additive genetic correlations of 0.49 and 0.89 between the two traits, respectively, in Sarda ewes. These authors also described favorable (lactose) and unfavorable (SCS) phenotypic and genetic correlations with effects on both coagulation times and curd firming. In our study, a negative phenotypic correlation of 0.46 was found between these two traits (Supplementary Table S1). This is an important issue since Assaf sheep milk is mostly used to make cheese. Lactose is the most important osmotic regulator in milk and is very constant in milk samples [46]. In this sense, inflammation of the mammary gland can lead to an increased influx of ions from blood to milk and conductivity and decreased lactose content in high SCC milk [47].

## 5. Conclusions

The results reported in the present study suggested that two different regions of OAR19 influence the SCS. Although none of them were reported in previous studies, it should be kept in mind that resistance to mastitis is a polygenic and complex trait that is difficult to explain considering only a few markers. Further studies will be needed to isolate the functional SNPs responsible for the phenotypic variation observed for the SCS trait in this study.

## Figures and Tables

**Table 1:** Corrected phenotype estimates mean values and standard deviation for the somatic cell score (SCS) trait for the animals selected for the genome-wide association study (GWAS) within high and low SCS tails. The ewes selected for these groups were in the highest 10% and lowest 14% tails of the distribution of estimated corrected phenotypes obtained for all ewes (*n* = 1894).

Population	LOW SCS	HIGH SCS
Flock A (*n* = 64)	−0.77 ± 0.09	1.61 ± 0.41
Flock B (*n* = 64)	−0.91 ± 0.14	2.15 ± 0.38
Flock C (*n* = 64)	−0.72 ± 0.04	1.79 ± 0.54
Total (*n* = 192)	−0.80 ± 0.13	1.85 ± 0.50

**Table 2:** MLMA results for significant SNPs at the chromosome level (FDR *p* < 0.10) for the estimated somatic cell score (SCS) trait. The SNPs are ordered according to their positions in the Oar 3.1 genome version (Ensembl release 81). Minor allele frequency (MAF) refers to allele 1 (A1).

Chr^1^	SNP	Oar3.1	A1	A2	MAF	b	se	*p*-Val	*p*-Val_FDR10	Genes_250Kb
19	rs415580501	9401922	C	A	0.26	0.71	0.17	3.98 × 10^−5^	4.00 × 10^−5^	*ARPP21-miR128_2*
19	rs410336647	9407458	G	A	0.26	0.71	0.17	3.98 × 10^−5^	4.00 × 10^−5^	*ARPP21-miR128_2*
19	rs424642424	9410968	A	G	0.26	0.71	0.17	3.98 × 10^−5^	4.00 × 10^−5^	*ARPP21-miR128_2*
19	rs419096188	58334807	A	G	0.38	−0.61	0.15	2.91 × 10^−5^	4.00 × 10^−5^	*FBLN2-Ensoarg00000001587-Ensoarg00000026664-HDAC11-NUP210-Ensoarg00000026665-* *Ensoarg00000026666*

^1^ Chr: chromosome, SNP: reference SNP ID number, Oar3.1: position in sheep genome version Oar3.1, A1: allele 1, A2: allele 2, b: allele substitution value, se: standard error, *p*-Val: *p*-value, *p*-Val_FDR10: the threshold for chromosome level (FDR 0.10), Genes_250Kb: annotated genes located in the 250 kb region on both sides of the significant SNPs.

**Table 3:** Minor allele frequency (MAF), type III test of the fixed effects and covariates, and lsmeans for the genotype effect of the SNPs rs419096188 and rs424642424 for the somatic cell score (SCS), milk yield (MY; mL), fat (FC; %), protein (PC; %), lactose (LC; %) and total solid content (TSC; %) traits. The number of animals for each genotype is indicated in brackets. Different letters indicate significant differences after Bonferroni correction: a, b: *p* <0.05; c, d: *p* <0.01.

SNP	MAF^1^	Trait	*p* -Values						LSmeans Genotype Effect		
			Htd^2^	DIM	Nlb	A	LN	G	AA	AG	GG
**rs419096188**	0.40	*SCS*	<0.001	<0.001	0.549	0.252	0.071	0.003	2.70 ± 0.10a,c (*n* = 282)	2.91 ± 0.08b (*n* = 893)	2.96 + 0.09a,b,d (*n* = 649)
		*MY*	<0.001	<0.001	<0.001	0.884	<0.001	0.337	2309.87 ± 74.02	2361.59 ± 58.98	2284.92 ± 63.53
		*FC*	<0.001	<0.001	0.007	<0.001	<0.001	0.204	5.43 ± 0.09	5.53 ± 0.07	5.47 ± 0.08
		*PC*	<0.001	<0.001	0.632	0.730	<0.001	0.755	5.27 ± 0.04	5.28 ± 0.03	5.26 ± 0.03
		*LC*	<0.001	<0.001	0.035	0.138	<0.001	0.015	4.83 ± 0.02a	4.81 ± 0.02a,b	4.78 ± 0.02b
		*TSC*	<0.001	<0.001	0.528	0.317	0.546	0.075	11.03 ± 0.04	11.01 ± 0.03	10.97 ± 0.04
**rs424642424**	0.25	*SCS*	<0.001	<0.001	0.515	0.289	0.088	0.009	2.92 ± 0.13a,b (*n* = 103)	2.99 ± 0.09a (*n* = 689)	2.82 ± 0.08b (*n* = 1031)
		*MY*	<0.001	<0.001	<0.001	0.787	<0.001	0.144	2081.7 ± 135.31	2331.85 ± 61.34	2336.26 ± 57.33
		*FC*	<0.001	<0.001	0.007	<0.001	<0.001	0.158	5.59 ± 0.12	5.54 ± 0.08	5.46 ± 0.07
		*PC*	<0.001	<0.001	0.643	0.720	<0.001	0.505	5.29 ± 0.05	5.28 ± 0.03	5.26 ± 0.03
		*LC*	<0.001	<0.001	0.033	0.183	<0.001	0.929	4.80 ± 0.03	4.80 ± 0.02	4.80 ± 0.02
		*TSC*	<0.001	<0.001	0.502	0.359	0.625	0.719	11.00 ± 0.05	11.01 ± 0.04	10.99 ± 0.03

^1^MAF: minor allele frequency; ^2^Htd: herd-test day, DIM: the days in milk, Nlb: number of lambs born, A: age, LN: lactation number, G: genotype.

## Data Availability

The data presented in this study are available on request from the corresponding author.

## Supplementary Materials

The following are available online at https://www.mdpi.com/article/10.3390/ani11061531/s1, Figure S1: Sample structure identified by multidimensional scaling analysis taking into account the three flocks (a) and the two groups of ewes with extreme animal estimated values for SCS (b), Figure S2: Manhattan (a) and Q-Q (b) plots from GWA analysis of estimated SCS trait in Assaf sheep. Chromosomes 1-26, and X (27) are shown separated. Green line in Manhattan plot corresponds to average threshold value for a FDR of 10% evaluated at chromosomal level, Table S1: Pearson phenotypic correlations for the somatic cell score (SCS), milk yield (MY; mL), fat (FC; %), protein (PC; %), lactose (LC; %), and total solid content (TSC; %) traits in the total population.
