# Supplementary material for: Genome-Wide Association Studies of Somatic Cell Count in the Assaf Breed
Source: Animals (Basel). 2021 May 24;11(6):1531. doi: 10.3390/ani11061531 (PMC8225172; doi:10.3390/ani11061531)

Supplementary Figure 1. Sample structure identified by multidimensional scaling analysis taking into account the three flocks (a) and the two groups of ewes with extreme animal estimated values for SCS (b).

a)

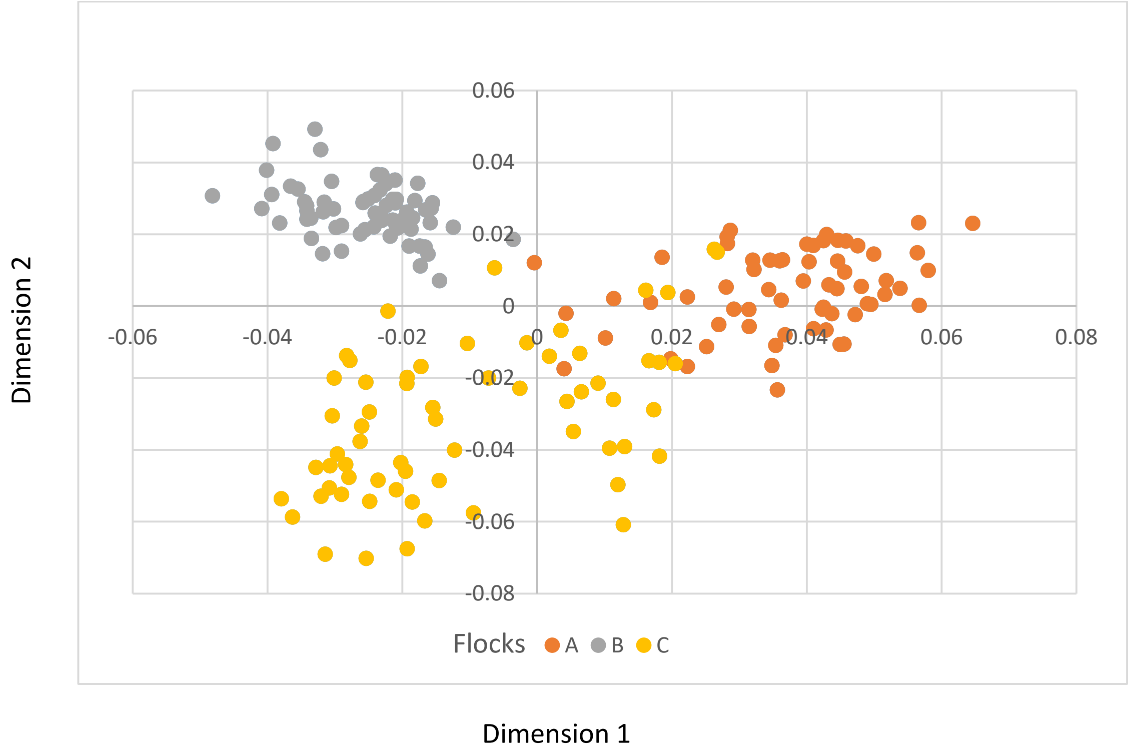

b)

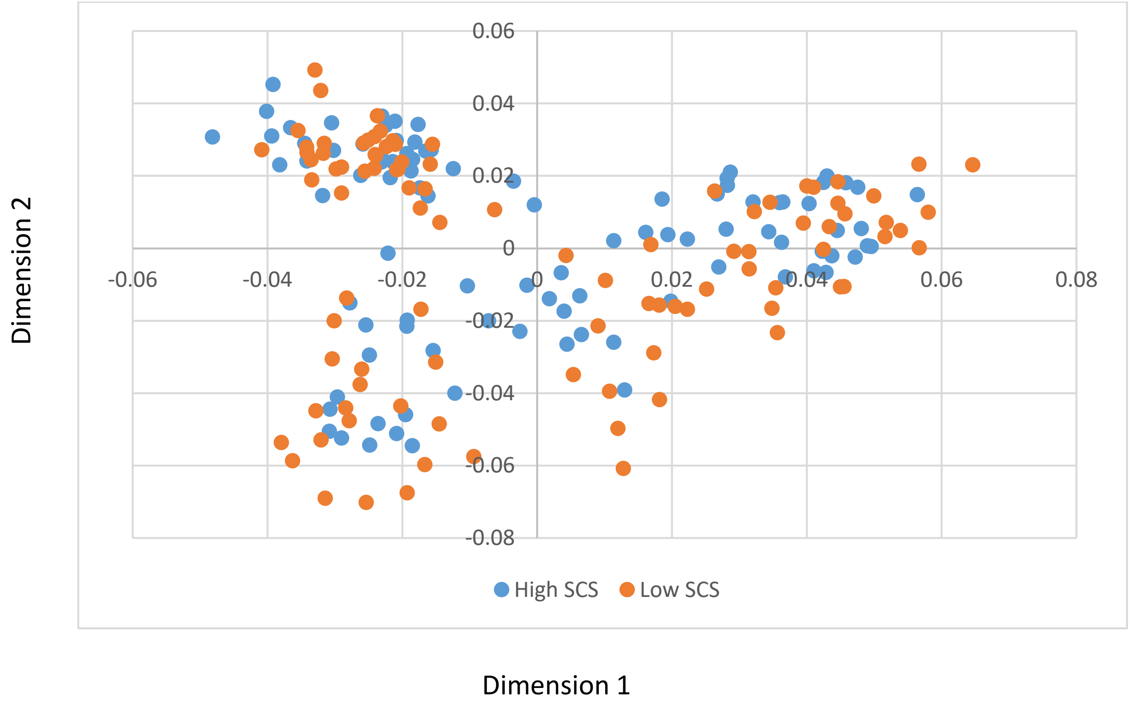

Supplement: Supplementary file 1 [file animals-11-01531-s001.zip › animals-1211644-supplementary/Supplementary Figure 1.pdf]
