# Supplementary material for: Genome-Wide Association Studies of Somatic Cell Count in the Assaf Breed
Source: Animals (Basel). 2021 May 24;11(6):1531. doi: 10.3390/ani11061531 (PMC8225172; doi:10.3390/ani11061531)
Supplement: Supplementary file 1 [file animals-11-01531-s001.zip › animals-1211644-supplementary/Supplementary Figure 2.pdf]

Supplementary Figure 2. Manhattan (a) and Q-Q (b) plots from GWA analysis of estimated SCS trait in Assaf sheep. Chromosomes 1-26, and X (27) are shown separated. Green line in Manhattan plot corresponds to average threshold value for a FDR of 10% evaluated at chromosomal level.

a)

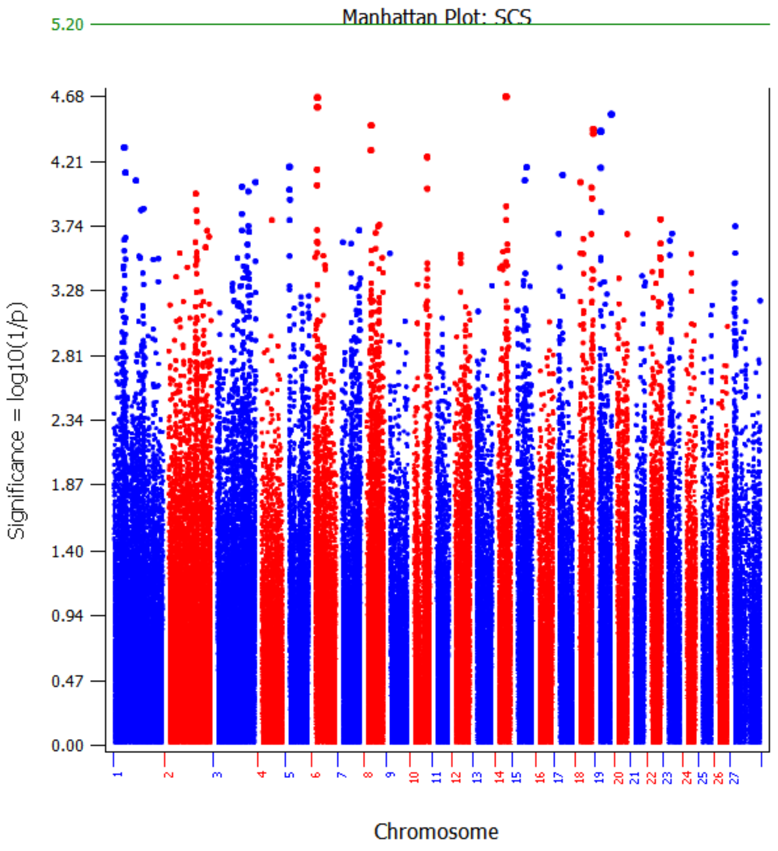

b)

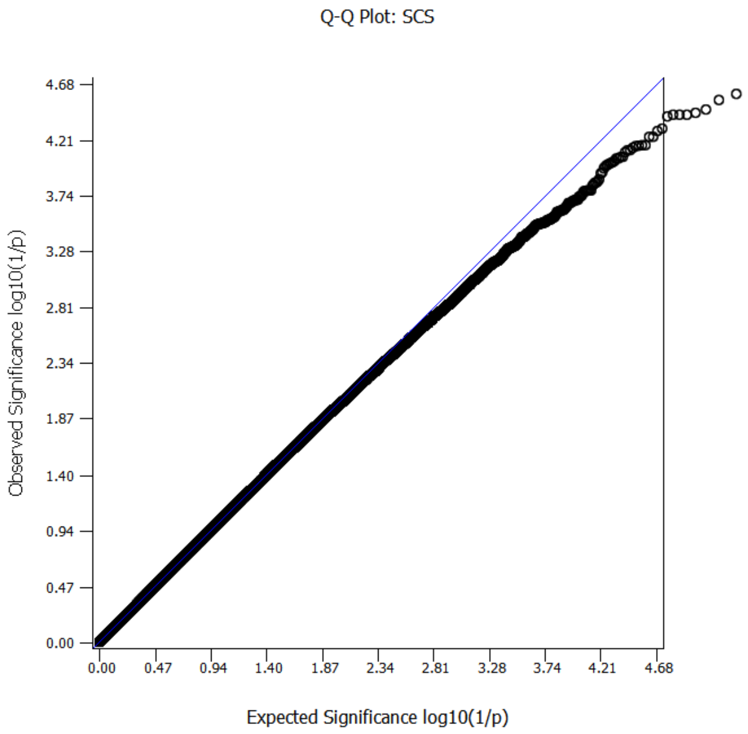
Q-Q Plot: SCS
Observed Significance log10(1/p)
Expected Significance log10(1/p)
4.68
4.21
3.74
3.28
2.81
2.34
1.87
1.40
0.94
0.47
0.00
0.00
0.47
0.94
1.40
1.87
2.34
2.81
3.28
3.74
4.21
4.68
