# Supplementary material for: Genome-Wide Association Studies of Somatic Cell Count in the Assaf Breed
Source: Animals (Basel). 2021 May 24;11(6):1531. doi: 10.3390/ani11061531 (PMC8225172; doi:10.3390/ani11061531)
Supplement: Supplementary file 1 [file animals-11-01531-s001.zip › animals-1211644-supplementary/Supplementary Table 1.docx]

**Supplementary Table 1.** Pearson phenotypic correlations for the somatic cell score (SCS), milk yield (MY; mL), fat (FC; %), protein (PC; %), lactose (LC; %) and total solid content (TSC; %) traits in the total population.

|  | **SCS** | **FC** | **PC** | **TSC** | **LC** |
| --- | --- | --- | --- | --- | --- |
| **FC** | 0.20 |  |  |  |  |
| **PC** | 0.19 | 0.66 |  |  |  |
| **TSC** | -0.08 | 0.45 | 0.83 |  |  |
| **LC** | -0.46 | -0.36 | -0.29 | 0.28 |  |
| **MY** | -0.19 | -0.49 | -0.37 | -0.17 | 0.39 |
